# Transcriptome Profiling Reveals PHLDA1 as a Novel Molecular Marker for Ischemic Cardiomyopathy

**DOI:** 10.1007/s12031-018-1066-6

**Published:** 2018-05-08

**Authors:** Jinhui Wang, Feifei Wang, Jingbin Zhu, Mei Song, Jinghong An, Weimin Li

**Affiliations:** 1 Clinical Laboratory, The First Hospital of Harbin, Harbin, Heilongjiang Province China; 2 Orthopedics, The First Hospital of Harbin, Harbin, Heilongjiang Province China; 3 Cardiology, The First Hospital of Harbin, Harbin, Heilongjiang Province China

**Keywords:** Ischemic cardiomyopathy, Apoptosis, PHLDA1, Microarray, Transcriptome

## Abstract

Ischemic cardiomyopathy (ICM) represents a worldwide health issue owning to its high sudden death rate. Easy diagnosis and effective treatment of ICM are still lacking. Identification of novel molecular markers will help illustrate the pathophysiology of ICM and facilitate its diagnosis and targeted treatment. Transcription profiling could be an easy and efficient way for identifying new markers. However, the mega data in the available database may contain a large number of false-positive hits. To identify the true marker for ICM, we systematically compared available microarray datasets in the GEO database and identified 26 genes that are shared by all datasets. We further verified the expression pattern of these 26 genes in ICM rat model. Only 12 genes show significant differential expression in our animal model. Among them, we focused on PHLDA1, a well-documented pro-apoptotic factor. Expression of PHLDA1 was elevated in both ischemic cardiac cell lines and in rat model. Overexpression of PHLDA1 promotes apoptosis of cardiac muscle cell. Meanwhile, PHLDA1 not only inhibited AKT pathway, but also activated p53 pathway. We thus confirmed PHLDA1 as a true molecular marker for ICM.

**Electronic supplementary material:**

The online version of this article (10.1007/s12031-018-1066-6) contains supplementary material, which is available to authorized users.

## Introduction

Ischemic cardiomyopathy (ICM) is the leading cause of sudden cardiac death worldwide (Candell-Riera et al. 2009). ICM is featured by narrowing of coronary arteries, myocyte death, reactive cellular hypertrophy, and ventricular scarring, which eventually lead to insufficient blood supply to the heart muscle and heart failure (Beltrami et al. 1994). Diagnosis of ICM heavily relies on the instrumental examination—quick and easy screening for ICM diagnosis is still lacking. Despite significant advances in ICM treatment, the morbidity and mortality remains high. Thus, identifying new molecular markers and drug targets are in urgent demand.

Previous studies have revealed that multiple molecular signaling pathways in cardiac hypertrophy, inflammatory signaling, oxidative stress, and calcium signaling contribute to ICM (Feldman et al. 2005; Wehrens and Marks 2004; Yano et al. 2005). Moreover, stress-induced apoptosis is responsible for sustained cardiac cell death, cardiac dysfunction and ultimately heart failure (Fliss and Gattinger 1996; Foo et al. 2005). ICM therapies targeting the apoptosis pathway have been widely explored. Inhibitors targeting apoptotic signaling β-adrenergic receptor are commonly used in heart failure therapy (Nussinovitch and Shoenfeld 2013). Broad-spectrum caspase inhibitors have been shown to prevent dilation, attenuate cardiac dysfunction and reduce heart failure in transgenic mice (Wencker et al. 2003; Hayakawa et al. 2003; Chandrashekhar et al. 2004). Proteins involved in apoptosis signaling could be potential therapeutic targets or diagnosis markers for ICM.

To identify novel markers and targets, we turned to gene expression patterns associated with ICM, on the hypothesis that genes involved in ICM pathology will be differently expressed in ICM patients or animal models. Benefiting from technical advances in microarrays and RNA sequencing, global analysis of gene expression profiles can be performed. Such screenings have been used to search for novel markers or targets for cardiomyopathy (Asakura and Kitakaze 2009; Nanni et al. 2006). However, deep mining and validation of gene expression data is necessary to isolate the true ICM markers and targets from false-positive hits. In this report, we systemically examined the available transcriptome datasets in the Gene Expression Omnibus (GEO) database. We found that 26 genes associated with ICM were reported to be differentially expressed in all datasets. In our rat ICM models, 12 out of 26 genes were with either significantly down- or up-regulated transcription. Among them, PHLDA1 shows increasing overexpression over time, and has been documented as promoting apoptosis (Neef et al. 2002; Park et al. 1996). PHLDA1 overexpression was further validated with immunohistochemical staining and western blot assay. We also found a similar extent of PHLDA1 overexpression in cardiac muscle cells under ischemia conditions. Then, we showed that the PHLDA1 overexpression can promote cardiac muscle cell apoptosis. Moreover, expression of PHLDA1 inhibited phosphorylation of AKT and activated the p53 signaling pathway. Thus, PHLDA1 could be a novel molecular marker for ICM. Our results demonstrate the feasibility of identifying new molecular markers or targets for ICM using data mining and experimental validation.

## Methods

### Reagents

Antibody against PHLDA1 was obtained from Invitrogen (#PA5-53889), antibodies against actin (#ab109193), phosphor-AKT (#ab81283), AKT (#ab182729), p27 (#ab193379), p53 (#ab26), bax (#ab32503), bcl-2 (#ab59348) were obtained from Abcam. All primers for Q-PCR were ordered from Hongxun (Suzhou, China).

### Cell Lines and Culture Condition

The human H9C2 cardiac muscle cell lines were maintained in DMEM medium in incubator with 5% CO_2_ at 37 °C.

### Rat ICM Model

All procedures were approved by the Animal Care and Use Committee of the First Hospital of Harbin (Harbin, Heilongjiang, China) and conducted in accordance with Animal Research Committee Guidelines. We created an ICM rat model with coronary artery ligation method. In brief, healthy specific-pathogen-free (SPF) male Wistar rats were purchased from Vital River. All rats were preserved under standard laboratory housing conditions. After 1 week of adaptation to the diet and the new environment, male Wistar rats with body weight between 250 to 350 g were anesthetized with 3% sodium pentobarbital. The rats were randomly divided into three groups of 15 rats each, with five rats in each cage: control and model groups. For the model groups, hair on the left chest was removed and an incision was made inintercostal space between third and fourth ribs. The chest was then opened to expose the heart. A small needle with a piece of suture was passed through the pathway of coronary artery to ligate the coronary artery. The chest incision and the skin incision were then closed. For the control groups, the hair on the left chest was removed and the chest was opened, without any handling of the coronary artery. The skin incision was closed just as with the model group. The process was carried out at strict aseptic conditions, and the electrocardiogram was recorded.

### Q-PCR

Total RNA was isolated from animal tissue samples using trizol method. RNA was reverse-transcribed using the PrimeScript RT Master Mix (B-Belife, China) according to the manufacturer’s instructions. The primers used in Q-PCR were listed in Table S3. The PCR amplifications were performed using SYBR Premix Ex Taq II (B-Belife, China). The expression level of each sample was internally normalized against that of the glyceraldehyde 3-phosphate dehydrogenase. The relative quantitative value was calculated using 2^−ΔΔCt^ method. Each experiment was performed in triplicate.

### Western Blot

Frozen samples were homogenized in ice-cold RIPA buffer with complete mini protease inhibitor cocktail (Roche Diagnostics Ltd., East Sussex, UK) and centrifuged, and protein concentrations in the supernatants were determined. An equal amount of protein samples was loaded onto 10% SDS-PAGE and then transferred to NC membranes. After blocking with 5% BSA for 1 h at room temperature, the membranes were incubated with primary antibodies at 4 °C overnight. Then, the membranes were incubated with horseradish peroxidase-conjugated secondary antibody (cwbiotech, China) for 1 h at room temperature. The protein bands were visualized by ChemiDoc XRS+ (BioRad, USA).

### Immunohistochemistry

Rat tissue samples were fixed and cut to 5 μm thick. PHLDA1 antibody was applied to the sections for 30 min and incubated overnight at 4 °C, and then shaken at room temperature for 30 min. Antibody binding was amplified using biotin and streptavidin HRP for 10 min each, and the complex was visualized using DAB. All sections were assessed microscopically for positive DAB staining.

### Cell Transfection

To upregulate the expression of PHLDA1, the human PHLDA1 full-length cDNA was amplified and inserted into the pLV vector, and a scramble sequence was inserted into the pLV vector as the control vector. For transfection, the cells were seeded into 6-well plates. When cell confluency reached 50%, pLV-PHLDA1, pLV-GFP, or control plasmid was separately transfected into the cells using lipo2000 according to the manufacturer’s instructions.

### Apoptosis Assay

The H9C2 cells transfected with pLV-PHLDA1, pLV-GFP, or control plasmid were collected and resuspended to density around 1 × 10^6^ cells/ml. Then 5 μl of fluorochrome-conjugated Annexin V was added to 100-μl cell. After 10 min incubation at room temperature, cells were spun down and resuspended in 1× binding-buffer. Then 5 μl PI staining was added. Cells were examined with flow cytometry.

### Cytotoxic Lactate Dehydrogenase (LDH) Activity Detection

LDH activities were determined using the appropriate kits (Nanjing Jian Cheng Biotech Co., Ltd., Nanjing, China) according to the manufacturer’s instructions.

### Statistical Analysis

All the continuous variables were expressed as mean ± standard deviation (SD). One-way ANOVA were performed to detect difference among the groups. Student’s *t*-test was used for the difference analysis. A *p* value of more than 0.05 was considered as statistically significant. SPSS 13.0 for Windows (SPSS, Armonk, NY, USA) software was used for all the statistical analyses.

## Results

### Gene Expression Datasets of ICM

We searched through GEO database and found four datasets that were associated with human ICM samples (Table S1 — see Electronic Supplementary Material) (Kittleson et al. 2005; Gronich et al. 2010; Kong et al. 2010; Hannenhalli et al. 2006). The four datasets all came from left ventricular tissues, with in total 38 samples from ICM patients. To identify the true target from these datasets, we isolated the genes that were shared in all datasets. There are 26 common genes that are differentially expressed comparing to normal, with ten genes upregulated and 16 downregulated (Table S2). All 26 genes show statistical significant difference in expression based on their original data, with *p*-value between 1.34E-17 and 2.50E-03. The expression levels of these genes are subjected to further validation.

### ICM Animal Model for Gene Expression Validation

**Fig. 1:**
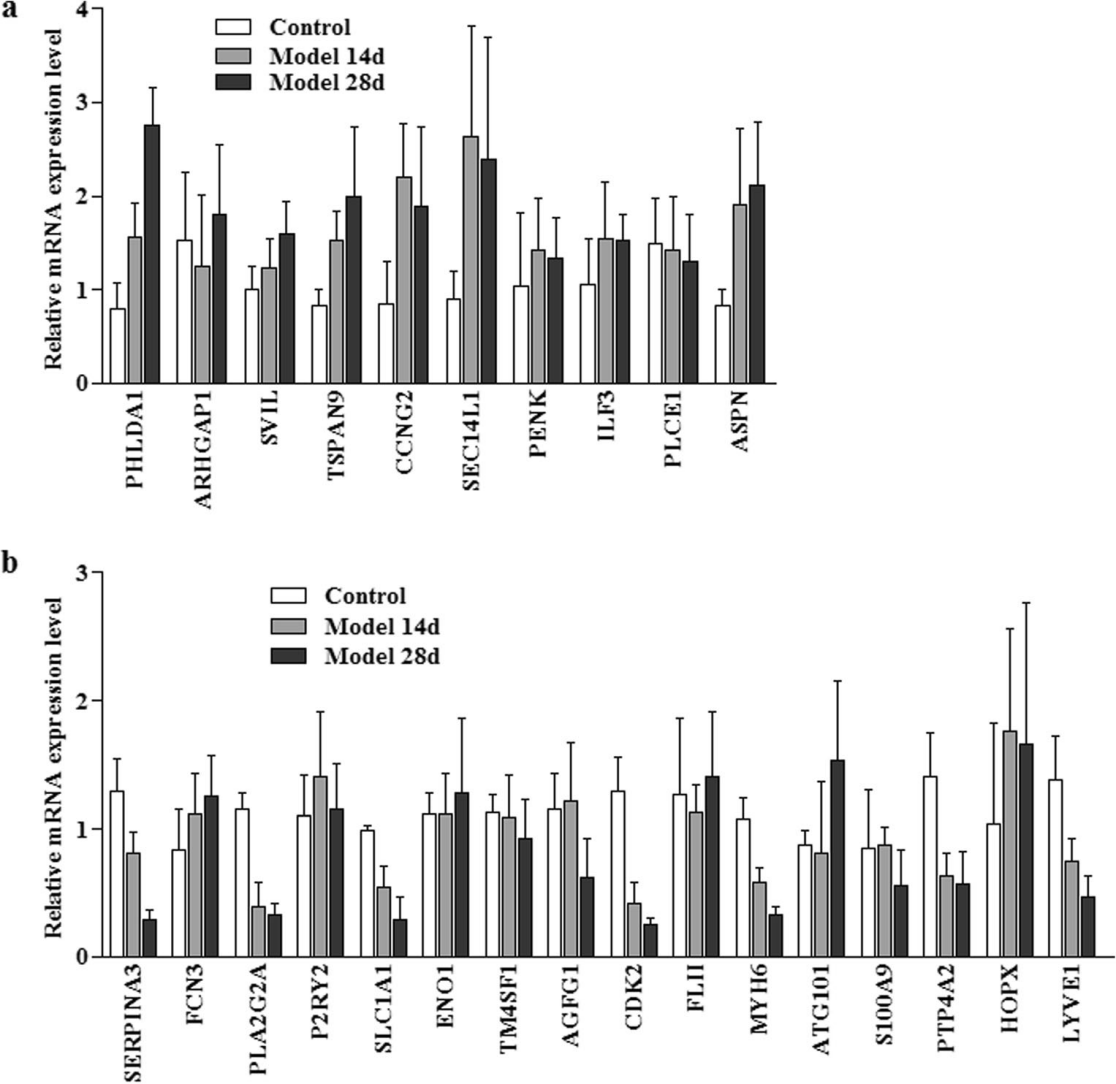
Expression level of genes in ICM rat model. Relative mRNA levels after 14 days and 28 days of coronary artery ligation were quantitated against control group. Each group contains three rats. **a** Genes reported to be upregulated in the microarray assays. **b** Genes reported to be downregulated in the microarray assays

We established rat ICM models using the coronary artery ligation method. To test gene expression, we took rat cardiac samples 14 days and 28 days after the surgery. The expression of the candidate genes were accessed by Q-PCR (Fig. 1). In our results, five genes show at least two-fold upregulation comparing to the control group, which are *PHLDA1*,* TSPAN9*,* CCNG2*,* SEC14L1*, and *ASPN*. The other five genes that reported to be upregulated show little difference in our assays. Seven genes, which were *SERPINA3*,* PLA2G2A*,* SLC1A1*,* CDK2*,* MYH6*,* PTP4A2*,and *LYVE1*, show clear down-regulation comparing to control group. Again, the remaining nine genes show little difference in their expression compared to the control group. Among the differentially expressed genes, PHLDA1 has been reported to promote apoptosis, and it has not yet been associated with ICM in the literature. Moreover, its expression level robustly increases after coronary artery ligation. We thus chose PHLDA1 for further studies.

### PHLDA1 Protein Level Is Increased in ICM Animal Model

**Fig. 2:**
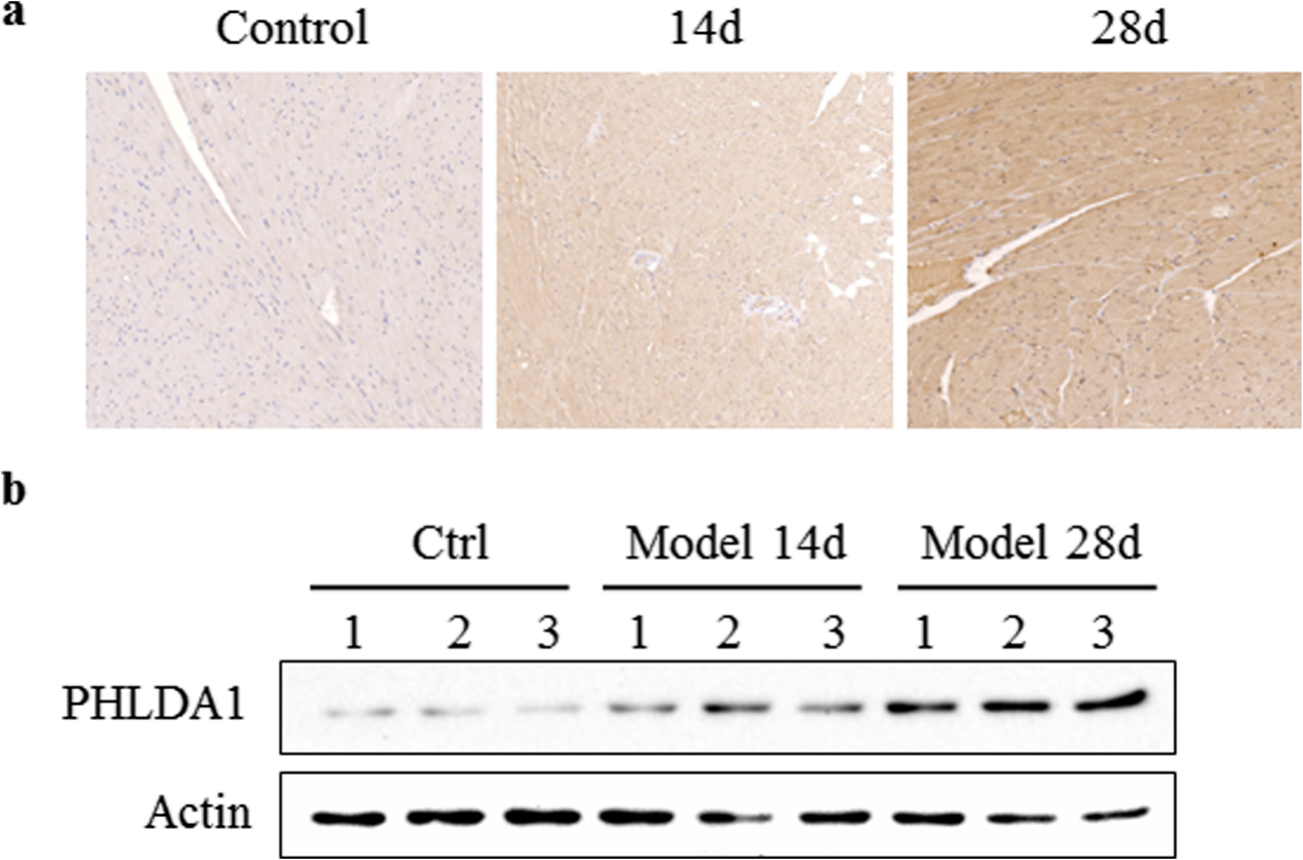
PHLDA1 protein expression level in ICM rat model. Left ventricular tissues from control rats (*left*) or ICM rats after 14 days (*middle*) and 28 days (*right*) of coronary artery ligation were tested by immunohistochemical staining (**a**) and western blot (**b**). The result indicated increasing protein level of PHLDA1. Each group contains three rats

As shown in Q-PCR, the *PHLDA1* level is increased by around three-fold in our rat model. We further tested whether the PHLDA1 protein level is also increased in our animal model. The staining of the animal tissue after 14 and 28 days of coronary artery ligation was by immunohistochemical staining and western blot. The results showed that PHLDA1 protein level was increased significantly after the surgery, as indicated by the increasing brown color in the staining (Fig. 2a) and elevated expression level in western blot assay (Fig. 2b).

### PHLDA1 Is Quickly Up-Regulated in Ischemia Cardiac Muscle Cell

**Fig. 3:**
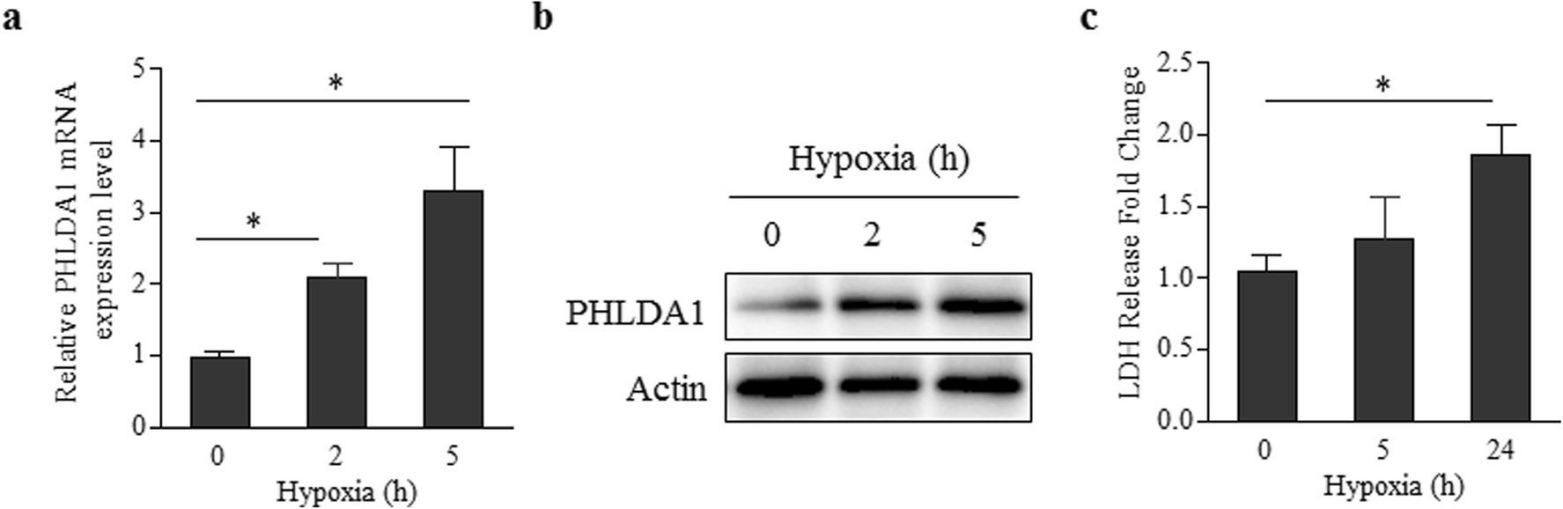
Expression of PHLDA1 and LDH release in ischemia cardiac muscle cells. **a** PHLDA1 mRNA expression. **b** PHLDA1 protein expression. **c** LDH release

To simulate ischemia conditions, we cultured the H9C2 cardiac muscle cell under hypoxia environment (0-5 h). The PHLDA1 expression was examined with Q-PCR and western blotting (Fig. 3). The results show that, similarly to the sample from our rat model, PHLDA1 was overexpressed in cardiac muscle cell. The *PHLDA1* mRNA level was around three-fold compared to control at 5 h (Fig. 3a). The PHLDA1 overexpression was validated by western blot, which show similar level of PHLDA1 overexpression (Fig. 3b). Meanwhile, the LDH activity was only 1.86 times higher comparing to control at 24 h (Fig. 3c). Collectively, these results indicated that PHLDA1 changed faster than LDH activity in ischemia cardiac muscle cell.

### Overexpression of PHLDA1 Promotes Cardiac Cell Apoptosis

**Fig. 4:**
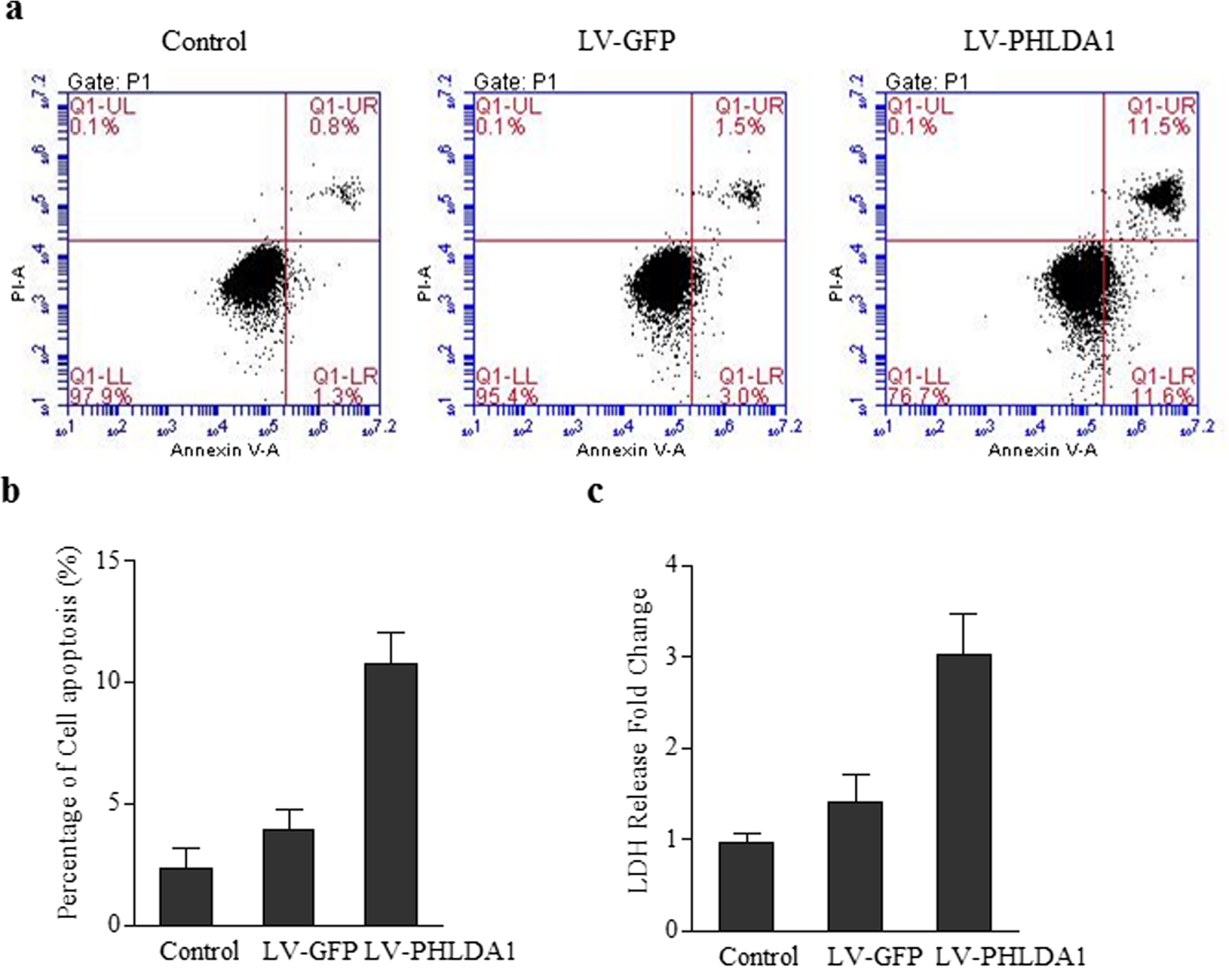
Overexpression of PHLDA1 induces apoptosis. **a** Annexin V/PI staining of cardiac cells without transfection, with GFP transfection, or with PHLDA1 transfection. **b** Respective summarized data are shown in the histogram. **c** LDH leakage activity of cardiac cells without transfection, with GFP transfection, or with PHLDA1 transfection

PHLDA1 has been reported to be involved in apoptosis signaling in T-cell and metastatic melanoma cells (Neef et al. 2002; Park et al. 1996). We tested whether PHLDA1 can induce apoptosis in cardiac muscle cells. The PHLDA1 gene in plasmid was transfected into the cardiac cell. The resulting cell line was subjected to Annexin and PI staining to quantitate the cell apoptosis. Without transfection or with GFP transfection, cardiac cells show only ~1% Annexin^+^/PI^+^. With PHLDA1 overexpression, the late apoptosis group (Annexin^+^/PI^+^) increased by at around 5-fold (Fig. 4a, b). We also tested the apoptosis with LDH leakage assay. The LDH activity was three times higher compared to control, while GFP plasmid showed a minor effect on LDH activity (Fig. 4c). The results demonstrated that PHLDA1 overexpression can promote cardiac cell apoptosis.

### PHLDA1 Affects AKT and p53 Pathway in Cardiac Cell

**Fig. 5:**
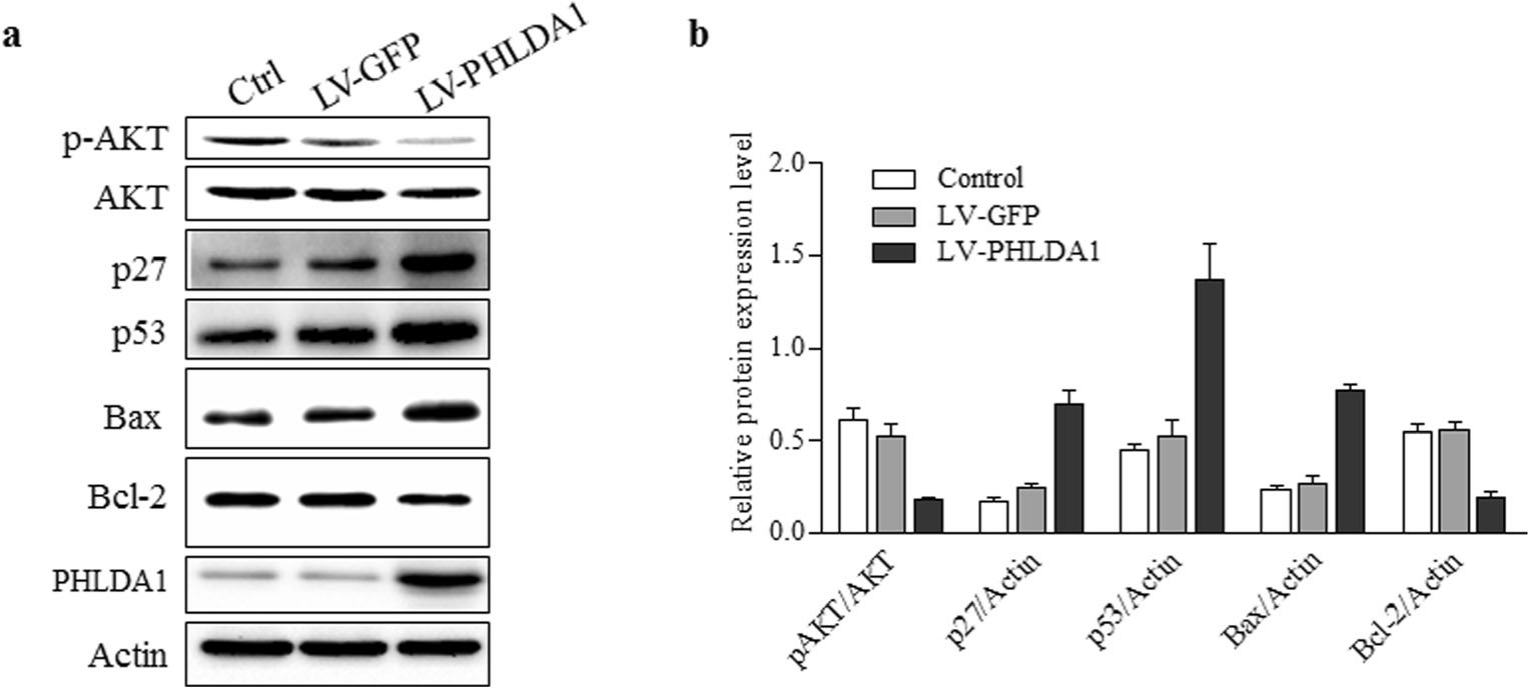
PHLDA1 expression reduced AKT phosphorylation and up-regulated p53 expression. **a** Western blot of cardiac cells without transfection, with GFP transfection or with PHLDA1 transfection. **b** Respective summarized data are shown in the histogram. Actin was used as control

Previous studies showed that AKT kinase phosphorylation increased in PHLDA1 downregulated cells (Li et al. 2014; Durbas et al. 2016), so next we evaluated the effects of PHLDA1 on AKT-p27 pathway in cardiac cells. As shown in Fig. 5a, b, PHLDA1 expression significantly reduced the phosphorylation of AKT and up-regulated p27 protein expression. Meanwhile, we detected the effect of PHLDA1 on p53, which is a well-known apoptosis-related signaling pathway. Results shown that PHLDA1 significantly increased p53 and Bax protein expression in cardiac cells, and inhibited Bcl-2 expression (Fig. 5a, b). These results help to explain how PHLDA1 promotes cardiac cell apoptosis.

## Discussion

ICM poses a worldwide health threat due to its high morbidity and mortality and its increasing occurrence in the aged population. Novel effective therapies are urgently needed. However, the detailed pathophysiology of ICM remains elusive. Traditional genetic analysis for screening genes related to disease could be effective but time- and labor-consuming. Advances in technique in genome sequencing and microarrays provide opportunities to check the global gene transcription patterns of patient samples. The analysis, when done properly, should yield insights of altered biological processes underlying diseases such as ICM. However, most of the transcription data have not been validated in cells or animal models. Whether the identified expression change under transcription level is real or not is still in question. Moreover, the underlying mechanisms for the transcriptional changes are not known, and the physiological consequences of the transcriptional changes are elusive. Thus, validations of the published datasets are necessary to identify true molecular markers or targets for ICM. The job is nontrivial, due to the large amount of data available.

In this report, we have thoroughly compared the four published microarray datasets from ICM patients (Kittleson et al. 2005; Gronich et al. 2010; Kong et al. 2010; Hannenhalli et al. 2006). In our analysis, only 26 genes were shared by all four datasets. Experimental validation with animal models further reduced the number of genes with significant differential expressions to 12, with five upregulated and seven downregulated. These genes only constitute a small portion of the original database. The huge difference among different datasets and between our experimental data could be due to different experimental conditions in different microarray assays, inconsistent selection criteria in diagnostic and inclusion criteria for patients and control groups, the difference between human patients and rat models, and false-positive hits from certain assay conditions. True biological markers and targets could be identified from microarray datasets; however, careful examination and experimental validation are absolutely needed.

We found 12 genes that are differentially expressed in both ICM patients and our animal models. Several of these 12 verified genes have been highlighted in literature for their relevance with ICM. MYH6 encodes a subunit of cardiac myosin (Matsuoka et al. 1989). Mouse model with knockout MYH6 is deficient in response to ischemia-reperfusion (Qi et al. 2014). In both patient samples and rat models, MYH6 is significantly downregulated, indicating its involvement in ICM development. CDK2 and CCNG2 encode cell cycle regulators, the activation of which possibly relate to myocardial apoptosis. Dominant-negative CDK2 expression can inhibit ischemia-induced apoptosis (Maejima et al. 2003). How the remaining genes would participate in ICM pathology is less well understood. Three extracellular proteins are in the final 12 genes: SERPINA3 encodes an extracellular protease inhibitor that is responsible for degradation and disassembly of myocardial protein (Hwang et al. 1999), ASPN encodes a cartilage extracellular protein that may regulate chondrogenesis and induce collagen mineralization (Ikegawa 2008), and SLC1A1 is a glutamate transporter (Kanai et al. 1995). Due to its extracellular localization, SERPINA3 has been suggested as a potential marker for ICM (Asakura and Kitakaze 2009). Several membrane proteins are in the 12 genes: TSPAN9 is a transmembrane protein that plays a role in the regulation of cell development, activation, growth, and motility (Protty et al. 2009), PLA2G2A is thought to participate in the regulation of the phospholipid metabolism (Kadam and Mulherkar 1999), and LYVE1 encodes an integral membrane glycoprotein that may function in lymphatic hyaluronan transport and have a role in tumor metastasis (Jackson 2003). PTP4A2 may function as a regulator of GGT II activity and have a role in tumorigenesis (Zhao et al. 1996). The function of SEC14L1 is unknown, and it has been suggested that orthologs of SEC14L1 may be involved in intracellular transport (Li et al. 2000). Further studies are needed to clarify roles of these genes in ICM. Finally, PHLDA1 is reported to be an apoptotic factor and has not yet been reported in ICM pathophysiology (Neef et al. 2002; Park et al. 1996). Considering the important role of apoptosis in sustained cardiac cell death and cardiac dysfunction, genes related to apoptosis such as PHLDA1 could be novel markers or targets for ICM.

PHLDA1 was first identified in T-cell receptor activation-induced apoptosis (Park et al. 1996). Following that, the pro-apoptotic role of PHLDA1 was verified in different cell lines (Neef et al. 2002). Due to its importance in apoptosis, PHLDA1 has attracted a lot of attention in cancer biology (Nagai 2016). Its abnormal expression has been linked to different cancers. However, there are no reports of PHLDA1 action in cardiac disease. Our results showed here that PHLDA1 is overexpressed in both patient samples and animal models. Moreover, the overexpression of PHLDA1 was also observed in cultured cardiac cells under ischemia. To confirm its pro-apoptotic role in cardiac cells, we tried overexpression of PHLDA1. As expected, high level of PHLDA1 can stimulate cardiac muscle cell apoptosis. In addition, PHLDA1 reduced the phosphorylation of AKT and activated the p53 signaling pathway. We thus conclude that PHLDA1 could play a pivotal role in cardiac cell apoptosis and ICM development. PHLDA1 is potentially a novel molecular marker and therapeutic target for ICM.

## Conclusion

Currently, gene expression profiles of cardiovascular disease have been performed. However, most of the data have not been validated in experimental model systems, hindering their usage in clinical and basic research. We systematically examined available transcriptome datasets of ICM and verified a portion of genes in our rat ICM model. Among them, PHLDA1, a reported pro-apoptotic protein, is connected to ICM. PHLDA1 overexpressed in patient samples, the rat ICM model, and cultured cardiac cells. Moreover, PHLDA1 overexpression leads to apoptosis of cardiac muscle cells via p53 pathway. Collectively, PHLDA1 is identified as a novel marker for ICM.

## Electronic supplementary material

ESM 1 (DOC 97 kb)
