# Supplementary material for: Transcriptome Profiling Reveals PHLDA1 as a Novel Molecular Marker for Ischemic Cardiomyopathy
Source: J Mol Neurosci. 2018 May 8;65(1):102–9. doi: 10.1007/s12031-018-1066-6 (PMC5978804; doi:10.1007/s12031-018-1066-6)
Supplement: Supplementary file 1 — (DOC 97 kb) [file 12031_2018_1066_MOESM1_ESM.doc]

**Transcriptome Profiling Reveals PHLDA1 as a Novel Molecular Marker for Ischemic Cardiomyopathy**

Jinhui Wang^1,4^, Feifei Wang^1,4^, Jingbin Zhu^2^, Mei Song^1^, Jinghong An^1^, and Weimin Li^3, *^

^1^ Clinical Laboratory, The First Hospital of Harbin, Heilongjiang Province, China.

^2^ Orthopedics, The First Hospital of Harbin, Heilongjiang Province, China.

^3^ Cardiology, The First Hospital of Harbin, Heilongjiang Province, China.

^4^ These authors contributed equally to this work

^*^ Correspondence: Weimin Li, wangadaw@gmail.com

**Supplement Tables**

Supplement Table 1. Gene expression datasets of ischemic cardiomyopathy from GEO database

| GEO accession | Author | Platform | Samples  (N:I) | Year | Tissue |
| --- | --- | --- | --- | --- | --- |
| GSE1869 | Kittleson MM | Affymetrix Human Genome U133A Array | 10:6 | 2005 | Left ventricular |
| GSE17294 | Gronich N | NIA_Human_Achannels 9k splice array | 3:2 | 2009 | Left ventricular |
| GSE16499 | Kong SW | Affymetrix Human Exon 1.0 ST Array | 15:15 | 2009 | Left ventricular |
| GSE5406 | Cappola TP | Affymetrix Human Genome U133A Array | 108:15 | 2006 | Left ventricular |

Supplement Table 2. The common 26 genes in ischemic cardiomyopathy compared to healthy control

| ID | Symbol | adj.P.Val | P.Value | logFC | Up/Down-regulated |
| --- | --- | --- | --- | --- | --- |
| 219087 | ASPN | 4.97E-14 | 1.34E-17 | 2.468 | up |
| 217996 | PHLDA1 | 8.59E-03 | 7.09E-06 | 1.707 | up |
| 213791 | PENK | 6.51E-03 | 2.49E-04 | 0.595 | up |
| 220968 | TSPAN9 | 1.46E-02 | 6.90E-05 | 0.56 | up |
| 211375 | ILF3 | 4.68E-02 | 2.50E-03 | 0.492 | up |
| 202084 | SEC14L1 | 3.97E-02 | 1.62E-03 | 0.47 | up |
| 202565 | SVIL | 2.52E-02 | 1.59E-03 | 0.427 | up |
| 205111 | PLCE1 | 1.45E-02 | 7.40E-04 | 0.373 | up |
| 202769 | CCNG2 | 3.61E-02 | 1.22E-03 | 0.336 | up |
| 218076 | ARHGAP1 | 2.01E-02 | 1.15E-03 | 0.167 | up |
| 205866 | FCN3 | 8.59E-03 | 6.24E-06 | -1.544 | down |
| 214468 | MYH6 | 4.36E-02 | 2.07E-03 | -1.493 | down |
| 211597 | HOPX | 2.35E-09 | 1.58E-12 | -1.487 | down |
| 202376 | SERPINA3 | 2.30E-13 | 8.25E-17 | -1.31 | down |
| 203649 | PLA2G2A | 2.02E-04 | 2.50E-06 | -1.261 | down |
| 220037 | LYVE1 | 7.25E-04 | 1.34E-05 | -1.041 | down |
| 203535 | S100A9 | 3.29E-02 | 2.35E-03 | -0.827 | down |
| 217294 | ENO1 | 9.99E-03 | 4.43E-04 | -0.789 | down |
| 208615 | PTP4A2 | 4.03E-05 | 2.88E-07 | -0.648 | down |
| 209387 | TM4SF1 | 2.11E-02 | 1.24E-03 | -0.469 | down |
| 211804 | CDK2 | 3.81E-02 | 1.46E-03 | -0.309 | down |
| 218214 | ATG101 | 4.15E-03 | 1.37E-04 | -0.301 | down |
| 213664 | SLC1A1 | 9.37E-03 | 4.07E-04 | -0.258 | down |
| 206277 | P2RY2 | 4.38E-03 | 1.49E-04 | -0.228 | down |
| 213926 | AGFG1 | 3.53E-02 | 1.17E-03 | -0.227 | down |
| 212024 | FLII | 1.60E-02 | 8.44E-04 | -0.204 | down |

Supplement Table 3. The primers in Q-PCR

| Gene | Primers (5'-3') |
| --- | --- |
| PHLDA1 | F:TCATCCACACCAACTCCAG |
|  | R:ATGCACTCTTCCCACTTCC |
| ARHGAP1 | F:CAGCAGGTTCTCCAAACTC |
|  | R:GGCAGATACCTGTACAAGGA |
| SVIL | F:CATAACTCCCATCTCATCCC |
|  | R:GCTTCAATATCTTCCAAGGGT |
| TSPAN9 | F:CATCGTCCTGTTGATCATCC |
|  | R:TTCTCGTTCACCTTGTCCA |
| CCNG2 | F:CAACAGCTACTACAGTGTTCC |
|  | R:CCAGAGTCCTCACTCTCAC |
| SEC14L1 | F:TCTGGACTCTGGTTAGTCCT |
|  | R:GGCCCTGGTAATCATTTCCT |
| PENK | F:CATCAACTTCCTGGCATGC |
|  | R:CAGGAGATCCTTGCAGGTC |
| ILF3 | F:TCCTTGACCATCCATCTGAC |
|  | R:GTTGACTGATAGCGTTTCTCC |
| PLCE1 | F:TATGTCAGCTTCTATCAGGAGG |
|  | R:TCTCCTACCACCAAACAACTC |
| ASPN | F:CGATTTGTTTCCGACATGC |
|  | R:ACTGATGTCAAACCTAGATCAG |
| SLC1A1 | F:ATGTCTGAGAACAAGACAAAGG |
|  | R:GAAGATAATCAGGCCCAGGA |
| TM4SF1 | F:CAGAAGGACCAAAGTGTAGC |
|  | R:AATCCAGAAGGTACTGTCCC |
| S100A9 | F:TAGCTTTGAGCAAGAAGATGG |
|  | R:CATACTTCCTAGAGTACTGATGG |
| PLA2G2A | F:CTGGAGAAACGTGGATGTG |
|  | R:CCTGGTTTGTAGAGCAGGA |
| ATG101 | F:GACTTCACATATGTGCGTGTC |
|  | R:CAGTGCATCCTTGAATTCCC |
| FLII | F:CTGGCTTACCTTCACATTCAG |
|  | R:TGTGTCATGCGTACTACCTC |
| PTP4A2 | F:ACTTTGGTCCGAGTTTGTG |
|  | R:AAATGGCCAGTCTAGAACG |
| P2RY2 | F:ATCCTCGTCACACCAAGAG |
|  | R:CTATTCCAGGAGTCCAGGC |
| ENO1 | F:CAAGGACTACCCAGTGGTG |
|  | R:GCAGTAGCTGTGAACTTCTG |
| HOPX | F:AGACGCAGAAATGGTTTAAGC |
|  | R:CTAGTCCGTGACCGATCTG |
| CDK2 | F:CTTGTTATCTCAAATGCTGCAC |
|  | R:TCCTGGAAGAAAGGGTGAG |
| MYH6 | F:GCAGACATAGAGACCTACCT |
|  | R:GTAGAAGATATGGTAGTTCCTCTC |
| ARFG1 | F:GACAGCGTGTTCAGTTCTG |
|  | R:TGTTCCAAAGACACTGCTG |
| LYVE1 | F:GGTTCTGGGACTGACTCTG |
|  | R:CATCCATAGCTGCAAGTCTC |
